# Inflammatory/Immune Adverse Events in Chronic Myeloid Leukemia Patients During Treatment With Bosutinib

**DOI:** 10.1002/cam4.70580

**Published:** 2025-02-05

**Authors:** E. Agostani, E. Tassistro, L. Antolini, C. Gambacorti‐Passerini

**Affiliations:** ^1^ Hematology Department Fondazione IRCCS San Gerardo Dei Tintori Monza Italy; ^2^ Department of Medicine and Surgery University of Milano‐ Bicocca Monza Italy; ^3^ Bicocca Center of Bioinformatics, Biostatistics and Bioimaging (B4 Centre), department of Medicine and Surgery University of Milano‐Bicocca Monza Italy; ^4^ Biostatistics and Clinical Epidemiology Fondazione IRCCS San Gerardo Dei Tintori Monza Italy

**Keywords:** bosutinib, CML, immune adverse events, SRC kinases

## Abstract

**Background:**

Bosutinib, a tyrosine kinase inhibitor (TKI), is effective in treating chronic myeloid leukemia (CML) patients resistant or intolerant to previous TKIs. Unlike other TKIs, bosutinib's lack of inhibition of c‐KIT and PDGFR may contribute to its unique tolerability profile. Similar to dasatinib, it targets Bcr/Abl and SRC kinases, particularly Lyn, raising safety concerns. In fact, the susceptibility of Lyn −/− mice to autoimmune disorders and the deregulation of Lyn‐dependent pathways in patients with lupus were previously shown.

**Aims:**

This study aimed to assess the time‐adjusted rate (TAR) of inflammatory/immune‐related adverse events in bosutinib‐treated patients.

**Methods:**

We analyzed clinical data from 60 patients with a minimum follow‐up of three months. We used the CTCAE dictionary to identify immune‐related adverse events (irAEs).

**Results:**

Patients had a median treatment duration of 47.9 months (IQR: 38.4–121.8), totaling 592.7 person‐months. Among 33 patients (55% of the sample), we detected 94 irAEs (2.3% of total adverse events), including giant cell arteritis, psoriasis, erythema nodosum, articular pain, pleural and pericardial effusion, and three cases of recurrent sterile pneumonia. The estimated TAR of the first irAEs was 14.7 (95% CI: 10.4–20.7) events per 100 person‐years; considering repeated irAEs, the TAR was 28.4 (95% CI: 23.2–34.8) events per 100 person‐years. The median time to the first irAE was 14.8 months (IQR: 7.1–42). These rates are higher than those observed in imatinib‐treated patients.

**Conclusions:**

Our findings support the clinical impression of a high incidence of irAEs in bosutinib‐treated patients and may lead to an enhanced understanding of bosutinib's safety profile and mechanism of action.

## 1 Introduction

Chronic myeloid leukemia (CML) is a myeloproliferative neoplasia with an incidence of approximately 2 new cases/10^5^ person‐years. The distinguishing element of this leukemia is the presence of a translocation between chromosomes 9 and 22 that results in the production of the Philadelphia chromosome (Ph) and of the Bcr‐Abl1 fusion oncoprotein [1].

Nowadays, the main therapy for chronic phase CML is based on Bcr‐Abl tyrosine kinase inhibitors (TKIs), such as imatinib, the first one introduced and still the most frequently used one [2]. Several other TKIs were introduced later on for patients with intolerance or resistance to the first‐generation drug [3]. Bosutinib, a third‐generation TKI, is a dual Bcr‐Abl and Src kinases inhibitor (particularly active against Lyn) without c‐KIT or PDGFR inhibitory activity [4, 5, 6].

In normal cells, the Src family of kinases (SFKs) are involved in immune response and they represent a critical part of the signaling mechanism [7]. In particular, Lyn, one of nine proteins belonging to this family, is found predominantly expressed in myeloid cells and in B lymphocytes. In these cells Lyn is associated with the B‐cell receptor, acting as a mediator in positive and negative signaling cascades. The initiation of inhibitory signaling cascades relies only on Lyn [8]. This Lyn‐dependent inhibitory pathway in mature B‐cells is fundamental for self‐tolerance and anergy. In fact, knock‐out mice for Lyn (Lyn ^−−/−−^) develop symptoms of autoimmune disease similar to human systemic lupus erythematosus (SLE), show hyperresponsivity to antigen stimulation and produce autoantibodies against many cytoplasmatic and nuclear components [9, 10]. According to this theory, there is evidence that a majority of patients suffering from SLE have reduced intracellular expression of Lyn inside B lymphocytes, even though this cannot explain the complex phenotype of the human disease [9, 11]. To further support the correlation between Lyn expression and immune‐related disorders, the onset of immune‐mediated‐thrombotic thrombocytopenic purpura and lupus‐like symptoms were observed in patients treated with dasatinib, another dual‐acting Bcr‐Abl/Src kinase inhibitor [12, 13].

Given the possible correlation between Lyn activity and autoimmunity, we sought to determine if patients treated with Bosutinib showed any adverse immune‐related (or inflammatory) effects.

## 2 Methods

This is a retro‐prospective observational cohort study in which data from patients enrolled in five different clinical studies performed at San Gerardo hospital (BOSEXTEND, BYOND, BFORE, SKI‐200 and SKI‐3000) were collected along with data from patients outside of clinical trials.

We included in the study only patients who were treated with bosutinib at our hospital, excluding those who were on treatment for less than 90 days since this period provides sufficient time to identify potential adverse events, while also minimizing the risk of confusing these events with signs and symptoms that may be related to the disease itself [14].

We also considered patients enrolled in some of the same clinical trials (SKI‐3000 and BYOND) but randomized to imatinib. These 10 patients, randomized to imatinib, were used as controls for the analysis of time‐adjusted rate (TAR).

We also included patients enrolled in the same clinical trials (SKI‐3000 and BYOND) who were randomized to imatinib. These 10 patients served as controls for the analysis of time‐adjusted rate (TAR).

**FIGURE 1:**
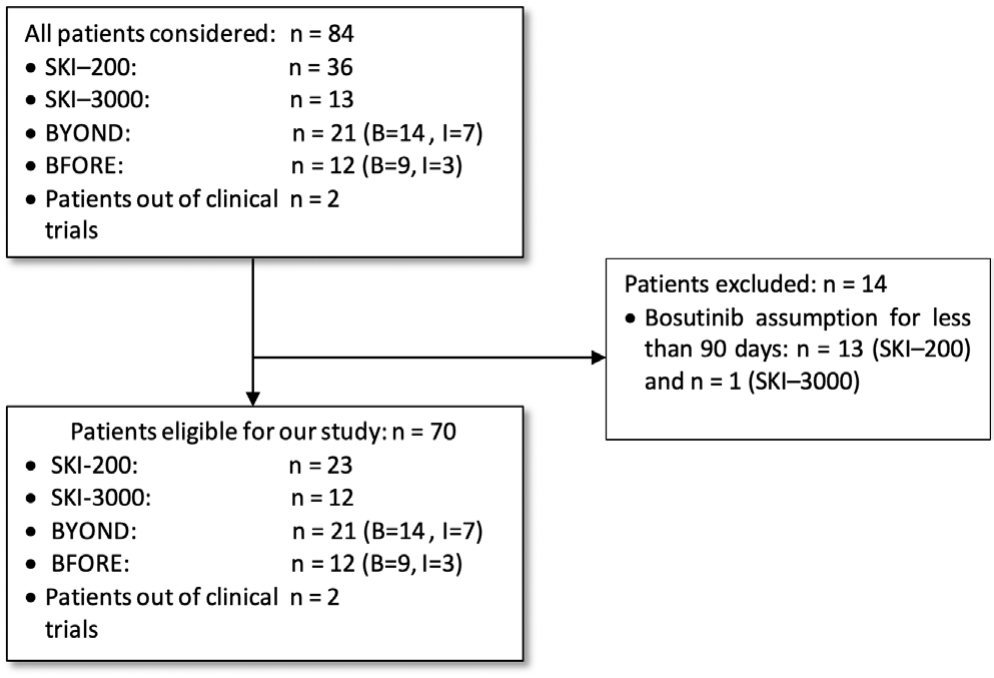
Flowchart of patients' selection process. B = bosutinib; I = imatinib.

A total of 60 patients on bosutinib and 10 patients on imatinib were selected, as shown in Figure 1.

Patient's data were collected from medical records. In particular, adverse events (AEs) were reported using the CTCAE terminology (v5.0).

For the purposes of this study, for every patient, these data were collected:

- Study of origin (only for patients in the studies);
- Identification code (ID code);
- Personal anamnestic data: date of birth and eventually death, gender, race, medical history;
- Primary diagnosis of leukemia and first diagnosis date;
- Prior therapies to bosutinib
- Bosutinib start date, and end date;
- Reasons for therapy interruption/discontinuation;
- AEs: denomination of the AE, onset date, grading, termination date, action undertaken to resolve the AE, causal relationship with the drug.

To identify immune‐related adverse events (irAEs) we draw up a list of terms, mainly using the CTCAE dictionary, to search in the complete database. We also included terms that do not exactly correspond to a diagnosis of autoimmune diseases but that more likely describe signs and symptoms of these disorders. For example, we included erythema nodosum but also erythematous papules and sub‐skin nodules. In addition, we considered valid for our analysis only those AEs for which a certain or possible relationship with bosutinib was indicated. We also included the term “fever,” when an infectious cause was ruled out.

In view of the above‐mentioned considerations, the list of terms utilized in the study includes AEs that are more likely to be considered inflammatory/immune rather than strictly autoimmune (See Table S1 in the Supplementary Appendix).

## 3 Statistical Analysis

Patients' characteristics were described through median and interquartile range (IQR) if the variables were continuous, and through frequencies and percentages if they were categorical. The comparison of the distribution of continuous and categorical characteristics across subgroups defined by the occurrence of AEs was obtained by the nonparametric test of the median and the chi‐square test, respectively. The distribution of the immune‐related adverse events (irAEs) was displayed through a barplot.

The occurrence of the first AE and repeated AEs was summarized through the calculation of the time‐adjusted rate (TAR) and of the 95% confidence intervals (CI) obtained by the exponential model.

The TAR of the first AE was obtained by the ratio of the number of patients who had a first AE and the total count of person‐years at risk. The contribution of the subjects who had a first AE was included in the count of the person‐years by the time of development of the first AE and the contribution of patients who did not have any AE by the available follow‐up time.

The cumulative incidence probability of the first AE by time “t” was estimated by the Kaplan–Meier method using the same definition of time at risk of the TAR of the first AE. To improve the interpretation, the incidence probability was then multiplied by 100 to interpret the result as the expected number of patients out of 100 who developed a first AE by the time “t.”

The TAR of repeated AEs was obtained by the ratio of the total number of events, accounting for the possibility of developing more than one AE on a single patient, and the total count of person‐years at risk. The contribution of the subjects in the count of the person‐years was included by the available follow‐up time even for patients who had at least one AE, to keep them at risk for the development of subsequent events. The impact of patient characteristics on TAR was obtained by the multivariable exponential survival model, being the hazard ratio of the exponential model equal to the ratio between time‐adjusted rates. This ratio is denoted by RR meaning rate ratio.

The expected number of AEs by time “t” on a single patient was estimated by the Aalen–Nelson method using the same definition of time at risk of the TAR of possibly repeated AEs. To improve the interpretation, the expected number of AEs on a single patient was then multiplied by 100 to interpret the result as the expected number of AEs observed by time “t” on 100 patients.

Statistical analyses were performed with Stata 15 software.

## 4 Results

### 4.1 Patients

A total of 60 patients with CP CML were selected for this study. Fifty‐nine (98.3%) subjects among them were Caucasian and 1 (1.7%) was Arabian. A total of 34 patients (56.6%) were males while the remaining 26 (43,4%) were females (Table 1).

**TABLE 1:** Patient characteristics.

Variable	Total (*n* = 60)	YES irAEs	NO irAEs	P value
Sex, n (%)				0.053
Male	34 (56.6)	15 (45.5)	19 (70.4)	
Female	26 (43.4)	18 (54.5)	8 (29.6)	
Age, (years)				
median (IQR)	62.8 (46.3, 70.3)	66.7 (52.9, 75.2)	54.9 (43.5, 68.1)	0.02
Follow‐up time, (months)				
median (IQR)	47.9 (35.7, 121.8)	44.8 (35.2, 70.7)	51.2 (43.4, 124.6)	0.126
Line of Bosutinib Therapy, n (%)				0.714
First line	14 (23.3)	7(21.2)	7 (25.9)	
Second line	32 (53.3)	17 (51.5)	15 (55.6)	
Third line	14 (23.3)	9 (27.3)	5 (18.5)	
Best response during bosutinib therapy (IS%)				
median (IQR)	0.01 (0.001, 0.06)	0.012 (0.001, 0.08)	0.007 (0.001, 0.02)	0.567

The median age of the 60 patients when they took the first dose of bosutinib was 62.8 (IQR 46.3–70.3) years, Patients who developed irAEs had a median age of 66.7 years while those who did not experience irAEs had a median age of 54.9 years (*p* = 0.02). The median bosutinib dose administered was 489.2 mg/day (IQR 437.1–509.6 mg/day) and the follow‐up, from the date of bosutinib start, was 47.9 (IQR 38.4–121.8) months.

IrAEs occurred in 33 subjects (55%, 18 females and 15 males) while 27 subjects (45%) presented no irAEs. Among the 60 patients who experienced irAEs, 14 (23.3%) received bosutinib as first‐line therapy, 32 (53.4%) as second‐line therapy, and 14 (23.3%) as third‐line therapy. All patients treated with bosutinib as a second‐line therapy (*n* = 32) had previously received a single TKI (imatinib in 31 cases and nilotinib in 1 case). For those treated with bosutinib as third‐line therapy, prior treatments included imatinib followed by dasatinib in 5 patients, imatinib followed by nilotinib in 2 patients, and interferon followed by imatinib in 7 patients. Moreover, the duration of previous treatments showed no statistically significant differences in relation to the occurrence of irAEs (*p* = 0.074) for the subset of 46 patients who had prior therapy lines. Among these patients, those without irAEs had a median treatment duration of 34.1 months, while those who experienced irAEs had a median treatment duration of 101.4 months (*p* = 0.126).

There were no significant differences in response levels to bosutinib between patients who experienced irAE and those who did not. The median response for patients without irAE was 0.007% International Scale (IS), while for those with irAE it was 0.012% IS. The p‐value of 0.567 indicates that the differences observed between the two groups are not statistically significant.

### 4.2 Adverse Events and Safety Profile

Immune‐related AEs constituted 2.3% (*n* = 94) of all registered adverse events (*n* = 4060).

**FIGURE 2:**
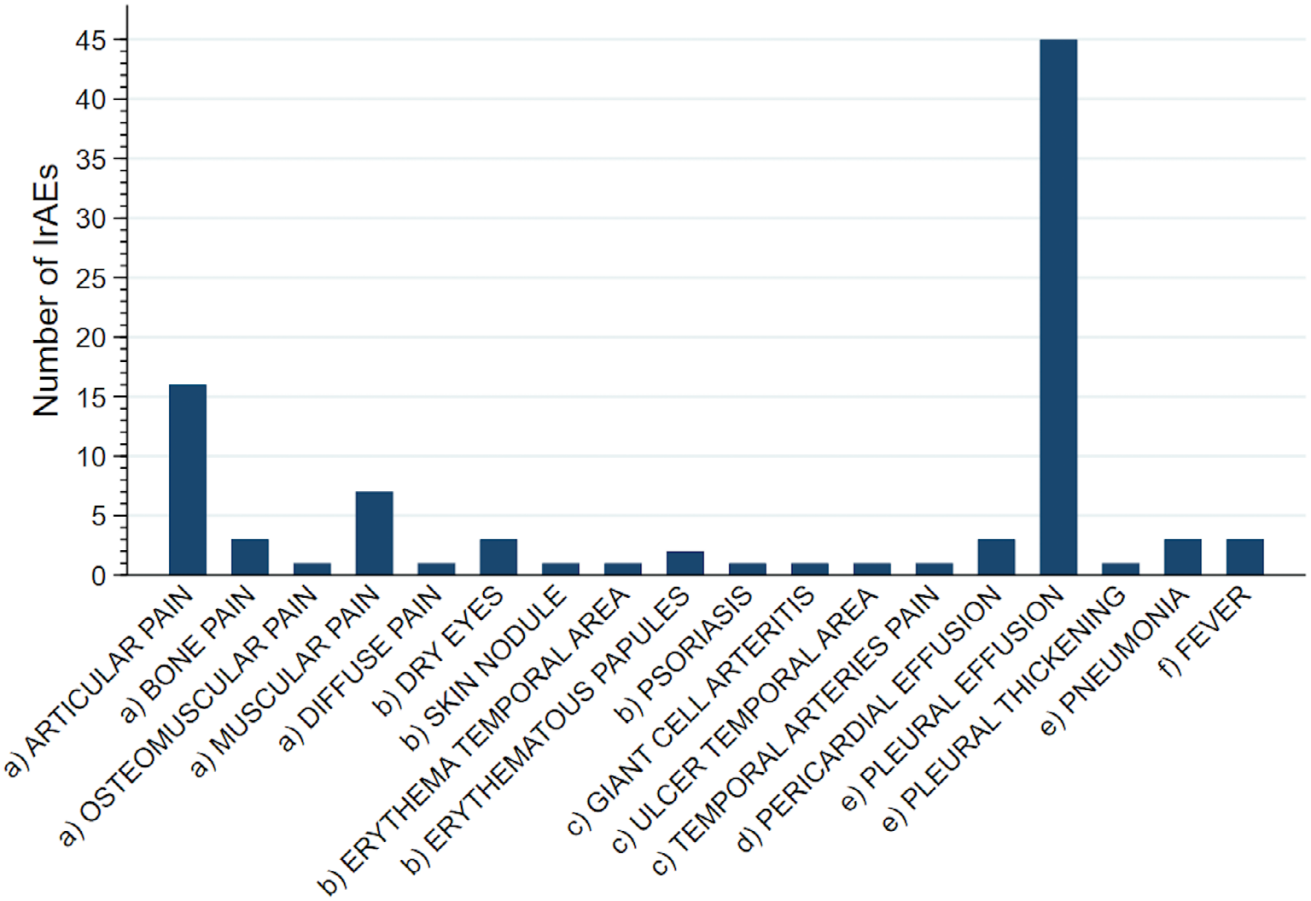
Immune‐related adverse events were observed in 60 subjects in different organs and systems. (a) Musculoskeletal system and connective tissue; (b) Skin and subcutaneous tissue; (c) Vascular system; (d) Heart; (e) Respiratory, thoracic, and mediastinal system; (f) General disorders.

These irAEs were divided into musculoskeletal and connective AEs, skin and subcutaneous AEs, vascular AEs, respiratory, thoracic and mediastinal AEs, cardiac AEs, and general AEs as shown in Figure 2. Pleural effusion was the most common irAE (*n* = 45) followed by articular pain (*n* = 16), muscular pain (*n* = 7), bone pain, dry eyes, pericardial effusion, pneumonia and fever (*n* = 3 each); erythematous papules occurred 2 times while osteomuscular pain, diffuse pain, pleural thickening, skin nodules, psoriasis, giant cell arteritis, erythema temporal area, ulcer temporal area, and temporal arteries pain occurred one time (Figure 2).

**FIGURE 3:**
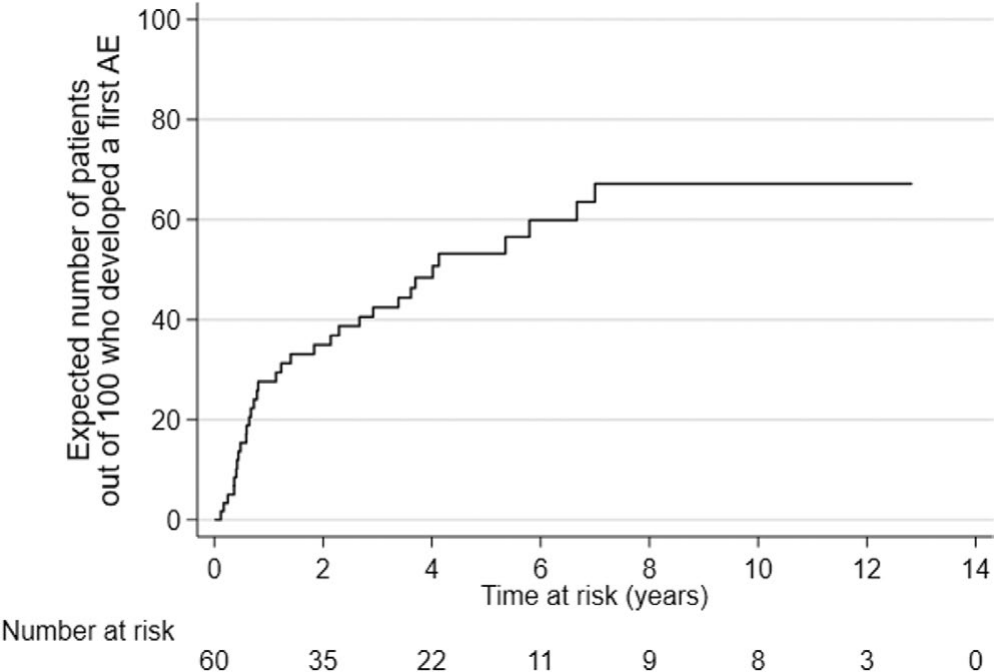
Cumulative probability to develop a first irAE.

Most irAEs (52.1%, *n* = 49,) occurred in the respiratory system, 30% (*n* = 28) in the musculoskeletal system and connective tissue, 8.5% (*n* = 8) in the skin and subcutaneous tissue, 6.4% (*n* = 6) in heart and vascular system. Non‐infectious fever occurred 3 times (3.2%). Pleural effusion (*n* = 45) occurred in 15 patients, 25% of all patients in our study.

Thirty‐three patients presented at least one irAEs as previously described. Considering only these 33 patients, an analytical description of them was presented in Table 2.

**TABLE 2:** Description of immune‐related adverse events.

ID	Provenience study	Organs and systems	Typology and number (*n*) of AE for each organ and system
01	SKI‐200	Musculoskeletal system and connective tissue	bone pain (1)
02	SKI‐200	Respiratory, thoracic, and mediastinal system	pleural effusion (2)
05	SKI‐200	Musculoskeletal system and connective tissue	articular pain (4)
		Skin and subcutaneous tissue	skin nodules (1)
		Respiratory, thoracic, and mediastinal system	pleural effusion (4)
08	SKI‐200	Heart	pericardial effusion (1)
09	SKI‐200	Respiratory, thoracic, and mediastinal system	pleural effusion (1)
11	SKI‐200	Respiratory, thoracic, and mediastinal system	pleural effusion (1)
12	SKI‐200	Respiratory, thoracic, and mediastinal system	pleural effusion (11)
15	SKI‐200	Respiratory, thoracic, and mediastinal system	pleural effusion (5)
18	SKI‐200	Respiratory, thoracic, and mediastinal system	pleural effusion (2)
21	SKI‐200	Respiratory, thoracic and mediastinal system	pleural effusion (9)
24	SKI‐3000	Musculoskeletal system and connective tissue	articular pain (1)
			muscle pain (1)
27	BOSEXTEND	Musculoskeletal system and connective tissue	articular pain (1)
28	SKI‐3000	Respiratory, thoracic, and mediastinal system	pleural effusion (1)
30	BFORE	Skin and subcutaneous tissue	psoriasis (1)
32	BFORE	Musculoskeletal system and connective tissue	articular pain (2)
33	BFORE	Musculoskeletal system and connective tissue	articular pain (1)
34	BFORE	Musculoskeletal system and connective tissue	articular pain (1)
		Skin and subcutaneous tissue	erythematous papules (2)
		Heart	
		Respiratory, thoracic, and mediastinal system	pericardial effusion (1)
			pleural effusion (1)
38	BYOND	Musculoskeletal system and connective tissue	dry eyes (1)
		Respiratory, thoracic, and mediastinal system	pleural effusion (1)
40	BYOND	Musculoskeletal system and connective tissue	articular pain (1)
42	BYOND	Respiratory, thoracic, and mediastinal system	pleural effusion (3)
43	BYOND	Musculoskeletal system and connective tissue	myalgia (1)
44	BYOND	Musculoskeletal system and connective tissue	articular pain (1)
46	BYOND	Musculoskeletal system and connective tissue	dry eyes (2)
49	BYOND	Heart	pericardial effusion (1)
50	BYOND	Musculoskeletal system and connective tissue	bone pain (2)
			myalgia (2)
51	BYOND	Musculoskeletal system and connective tissue	joint pain (1)
			osteomuscular pain (1)
			articular pain (2)
52	BYOND	Respiratory, thoracic, and mediastinal system	pleural effusion (1)
		General disorders	fever (3)
54	BYOND	Respiratory, thoracic, and mediastinal system	pleural effusion (1)
55	BYOND	Musculoskeletal system and connective tissue	articular pain (1)
			diffuse pain (1)
56	BYOND	Musculoskeletal system and connective tissue	muscular pain (2)
57	BYOND	Musculoskeletal system and connective tissue	muscular pain (1)
		Vascular system	giant cell arteritis (1)
			erythema temporal area (1)
			ulcer temporal area (1)
			temporal arteries pain (1)
59	Out of study	Respiratory, thoracic, and mediastinal system	pneumonitis (2)
60	Out of study	Respiratory, thoracic, and mediastinal system	pleural effusion (2)
			pneumonitis (1)
			pleural thickening (1)

Abbreviations: AE, adverse events.

All 60 patients treated with bosutinib, observed for 225 person‐years, originated 33 first irAEs. The estimated TAR for the onset of a first irAE was 14.7 (95% CI 10.4–20.7) events per 100 person‐years or 0.147 events per person‐years (Figure 3).

Considering repeated episodes of irAEs, overall the 60 patients at risk were observed for 330.89 person‐years and originated 90 irAEs. Therefore, the estimated TAR was 28.4 (95% CI 23.2–34.8) events per 100 person‐years.

**FIGURE 4:**
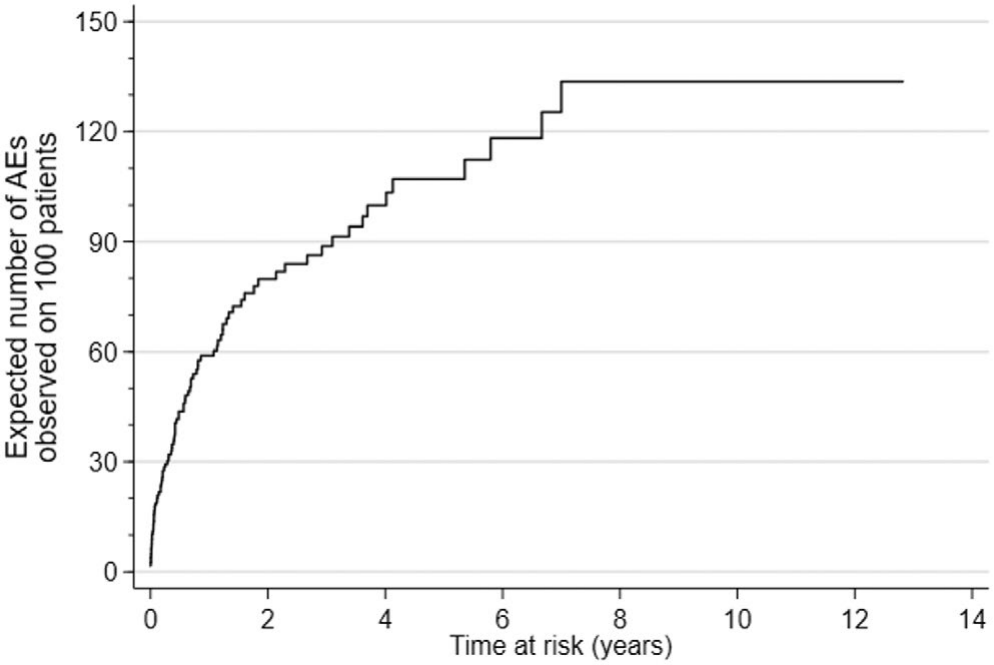
Expected number of irAEs observed in 100 patients.

The expected number of irAEs in 100 people was 59 at year 1, with an increase of 21 AEs at year 2. In the 7^th^ year, the total number of events reached 134 and stayed unchanged until the 12^th^ year, the maximum duration of the follow‐up for our cohort (Figure 4).

According to our data, the presence or absence of prior therapy lines before bosutinib did not have a significant impact on the rate of irAE. The comparison of irAE rates between patients with no prior therapy lines (TAR = 0.010; 95% CI: 0.005–0.021) and those with at least one prior therapy line (TAR = 0.013; 95% CI: 0.009–0.019) yielded a p‐value = 0.501, indicating no statistically significant difference.

After analyzing patient characteristics, we found that certain variables, such as age and gender, could impact irAE rates. We conducted a multivariate analysis to explore these associations further. The results showed that a 10‐year increase in age was significantly linked to a higher rate of irAEs (RR = 1.59, *p* = 0.001), while male patients had a significantly lower rate compared to females (RR = 0.43, *p* = 0.017). These findings highlighted the influence of age and gender on the incidence of irAE.

Analyzing the group of patients (*n* = 10) who were randomized in our clinical trials to receive imatinib, a total of 579 adverse events were observed, with 7 irAEs occurring in 4 patients. Considering the 10 patients at risk, they were observed for 47.65 person‐years and originated 4 first adverse events. The estimated TAR for the onset of a first inflammatory/immune AE was 8.4 events per 100 person‐years. The relative risk (RR) of developing an irAE in patients treated with bosutinib compared to those treated with imatinib was 1.75 (95% CI: 0.62–6.8, *p* = 0.29).

Considering repeated episodes of irAEs, overall the 10 patients at risk were observed for 49.86 person‐years and originated 6 irAEs. Therefore, in the imatinib group, the TAR was 12.01 events per 100 person‐years. The RR of recurrent irAE was 2.26 (95% CI: 0.99–6.32, *p* = 0.04) indicating a significantly higher risk of irAEs in the bosutinib group.

To better contextualize this study, it is useful to briefly expose some cases that aroused clinical suspicion about this category of bosutinib side effects (Table 3).

**TABLE 3:** Clinical manifestations of irAEs in five selected cases.

Patient	Sex	Age	Adverse Event	CRP and/or ESR	Autoimmunity tests	N° of episodes	Bosutinib dose start	Action taken	Outcome
# 34	F	70	Erythema nodosum	CRP 70.4 mg/L (n.r. 0–5) ESR 98 mm/h (n.r. 0–30)	ANA (neg) ANCA (neg) ENA (neg) Anti‐dsDNA (neg) C3 159 mg/dL (n.r 90–180) C4 38 mg/dL (n.r. 10–40)	1	400 mg	Rheumatological examination and corticosteroid therapy. Bosutinib discontinuation for 3 weeks and restart with dose reduction to 200 mg	Clinical and laboratory resolution
# 57	M	73	Horton arteritis	CRP 0.18 mg/dL (n.r. < 0.5); ESR 18 mm/h (n.r. 0–30)	RA test (neg)	1	500 mg	Rheumatological examination and corticosteroid therapy. Bosutinib permanently reduced to 200 mg	Clinical resolution
# 52	M	84	Pneumonia	CRP 3.25 (n.r. 0–5); ESR 110 mm/h (n.r. 0–30)	N/E	3	200 mg	BAL and blood cultures. Bosutinib discontinuation during all 3 episodes for 1 month. Restart with bosutinib 100 mg.	Clinical improvement (less intensity and rate of fever episodes)
# 59	F	79	Pneumonia	First episode: CRP 48.21 mg/L (n.r. 0–5) Second episode: CRP 71.14 mg/L (n.r. 0–5)	N/E	2	300 mg	Hospitalization, BAL, and blood cultures. Bosutinib discontinuation during the 2 episodes for 2 months. Restart with bosutinib 100 mg	Clinical improvement
# 60	F	82	Pneumonia	CRP 0.2 mg/L (n.r. 0–5)	N/E	2	200 mg	Hospitalization; antibiotic and corticosteroid therapy. Bosutinib discontinuation for 2 weeks during the first episode and for 1 months during the second episode. Restart with bosutinib 100 mg and then 200 mg.	Clinical improvement

Abbreviations: CRP, C‐Reactive protein; ESR, erythrocyte sedimentation rate; N/E, not evaluated; BAL, bronchoalveolar lavage; RA test, Reuma test; ANA, antinuclear antibody; ANCA, Anti‐neutrophil cytoplasmic antibody; ENA, extractable nuclear antigens antibody; anti‐dsDNA, Anti‐double‐stranded DNA.

Patient #34 (F) started bosutinib 400 mg in March 2015. Symptoms such as skin nodules, erythematous papules, and pain in lower legs, in addition to fever, started in July 2017. Since the symptoms did not resolve, the patient was seen by a rheumatologist who diagnosed Erythema Nodosum. Given the negativity of autoimmunity tests, he did not relate these symptoms to a primary autoimmune disease but linked this abnormal reaction of the immune system to an unidentifiable stimulus, possibly bosutinib. Systemic corticosteroid therapy was administered with the resolution of the clinical manifestation. During the treatment, there was a temporary suspension of bosutinib for 3 weeks, followed by a resumption of therapy at a reduced dose of 200 mg. After this adjustment, no further clinical relapses were observed.

Patient #57 (M) started bosutinib in November 2015 and developed headache, erythema in the right temporal area, and blurred vision since June 2016. The diagnosis of Horton arteritis was confirmed by a rheumatologist in September 2016 after a Doppler ultrasound of the temporal arteries. The patient started corticosteroid therapy with good control over inflammatory indices (CRP, ESR, RA‐test always negative) and symptoms were slowly resolved over a 3‐year period and did not recur thereafter. Following the diagnosis of arteritis, bosutinib was permanently reduced to 200 mg from 500 mg.

In Table 3, we also reported three patients (#52, #59, #60) who developed recurrent episodes (2 episodes in two cases and 3 episodes in the third one) of fever, cough, and lung infiltrates with subsequent diagnosis of pneumonia with no clear infectious etiology.

In all three cases, fever (38–39–) was uniformly present but microbiological tests (BAL and viral, bacterial, and fungal blood cultures) were persistently negative; CRP was 10–15– times the upper limit of normal (ULN) in patient #59 but in the other two patients it was negative. However, in patient #52, an increase in ESR was recorded. Two patients developed pneumonia with a bosutinib dosage of 200 mg and one patient with 300 mg. In all the three cases, symptoms disappeared after bosutinib was temporarily suspended and/or corticosteroid therapy was introduced. Upon reintroduction of bosutinib symptoms reappeared, but disappeared when bosutinib dosage was reduced to 100 mg in patient #52.

As already mentioned, the most frequent irAEs was pleural effusion which occurred in 15 patients. Of the 15 patients who experienced pleural effusion while on bosutinib, 10 temporarily stopped treatment for a median duration of 18 days, with resolution of the event. Among these, 6 patients resumed treatment at a reduced dose for a median duration of 6.9 months, while 4 resumed at the same dose as prior to the interruption. The remaining 5 patients did not interrupt treatment but instead managed the pleural effusion by reducing their bosutinib dosage for a median duration of 3.2 months. In all of these cases, the dose reduction resulted in effective management of both the adverse event and CML.

## 5 Discussion

Long‐term side effects from TKIs are very important to consider: patients enjoy a life expectancy similar to their peers, but treatment needs to be continued for many years.

Bosutinib is generally considered a safe TKI, with a remarkable long‐term safety profile, including cardiovascular AEs [15]. Bosutinib in particular does not cause peripheral lymphocytosis, at difference with dasatinib, another Abl/Src inhibitor. The prevalence of pleural/pericardial effusions, a hallmark of dasatinib‐related AEs, is much smaller when using bosutinib.

In an increasingly wide panorama of safety profile trials, our study is the first one designed to estimate the amount of inflammatory, possible autoimmune AEs (irAEs) in patients treated with bosutinib.

Bosutinib is a potent inhibitory of SFKs and in particular of Lyn. Given that Lyn knockout mice develop lupus‐like symptoms, it is reasonable to think that the disruption of Lyn activity might induce autoimmune disease, as also supported by evidence in human SLE patients [11].

We acknowledge the limitations in defining irAEs, particularly the inclusion of both specific autoimmune diseases and broader terms. This challenge arises from using standardized guidelines to classify events so, wherever possible, we contextualized these terms within each patient's clinical history to minimize misinterpretation and ensure the most accurate classification.

Over 50% of patients in our cohort experienced at least one irAE, with some experiencing recurrent irAEs. The time‐adjusted rate (TAR) of irAEs was 15 events per 100 person‐years for the first irAE and 28 events per 100 person‐years when considering recurrent irAEs.

When compared with imatinib‐treated patients, although the small sample size (*n* = 10) limits the strength of this comparison, the TAR for the first irAE was 8.4 per 100 person‐years (*p* = 0.29), and 12.01 per 100 person‐years for recurrent irAEs (*p* = 0.04). Although these findings suggest a higher incidence of irAEs in bosutinib‐treated patients, the small sample size of the imatinib group limits the strength of this comparison. Moreover, the statistical difference is significant only when considering recurrent adverse events, highlighting the need for cautious interpretation regarding the overall risk of irAEs in bosutinib‐treated patients.

There are several studies on bosutinib safety and tolerability, but no one presents adequate data for comparison. Dasatinib, the other dual Src/Bcr‐Abl inhibitor, has been in use for almost 15 years. However, we found no evidence in the literature regarding studies showing the time‐adjusted rate of irAEs. Several phase II and phase III studies assessed bosutinib and dasatinib safety, together with efficacy. The rate of AEs in these trials is expressed as an absolute frequency of patients that experienced the events, making the comparison with our TAR data difficult. In addition, these studies did not signal, not even in absolute value, the irAEs.

Therefore the present manuscript constitutes the first description of irAEs linked to bosutinib usage.

Importantly, our analysis of prior therapy lines and their duration before bosutinib initiation revealed no evidence of cross‐intolerance with bosutinib in patients previously treated with other TKIs. Moreover, the rates of irAEs remained consistent regardless of prior treatments, indicating that previous TKI exposure does not significantly influence bosutinib's tolerability.

We further investigated potential associations between patient characteristics, such as age and gender, and the incidence of irAEs. Multivariate analysis revealed that a 10‐year increase in age was significantly associated with a higher rate of irAEs, while male patients exhibited a significantly lower rate of irAEs compared to females. These results underscore the influence of age and gender on irAE incidence and highlight the importance of considering these factors in patient monitoring.

Finally, regarding the impact of irAEs on treatment efficacy, the median molecular response for patients without irAEs was 0.007% IS, while for those with irAEs, it was 0.012% IS. The p‐value indicates no statistically significant difference between the two groups, suggesting that irAEs do not substantially affect the efficacy of bosutinib. This finding aligns with the notion that the presence of irAEs may not interfere with the response to therapy.

Although a definitive conclusion regarding immune system‐related adverse effects has not yet been reached, we observed a high incidence of immune‐related autoimmune events, necessitating further confirmatory studies.

We recognize that this study is far from conclusive, and additional research is required to validate these findings and improve our understanding of the long‐term effects of bosutinib treatment. Given that TKI therapy for CML is often lifelong, continuous monitoring of long‐term adverse events, including autoimmune reactions, is critical, as they may emerge over time. While these adverse events may not pose an immediate life‐threatening risk, they can significantly impair quality of life and, consequently, therapeutic adherence. Our findings underscore the importance of closely monitoring irAEs during bosutinib treatment, as early intervention strategies could enhance patient management and help maintain a quality of life comparable to that of the general population, whose life expectancy is similar to that of CML patients on treatment.

## Author Contributions

Elena Agostani: Data curation – Lead, Formal analysis – Supporting, Investigation B – Equal, Writing – original draft – Lead. Elena Tassistro: Formal analysis – Lead, Software – Equal. Laura Antolini: Data curation – Supporting, Formal analysis – Equal, Software – Equal, Supervision – Equal. Carlo Gambacorti – Passerini: Conceptualization – Lead, Investigation – Equal, Project administration – Lead, Supervision – Lead, Writing – original draft – Supporting.

## Ethics Statement

This study did not require approval from an ethics committee, as it utilized publicly available, anonymized data and did not include any human or animal interventions. It was conducted in accordance with the ethical standards outlined in the Declaration of Helsinki. All referenced studies involving human participants were approved by the respective regional ethics committees.

## Consent

This study did not require the collection of informed consent from patients, as it utilized only anonymized data and did not involve direct interventions or new data collection. However, all referenced studies involving human participants obtained informed consent from their participants in accordance with their respective protocols.

## Conflicts of Interest

The authors declare no conflicts of interest.

## 6 Permission to Reproduce Material From Other Sources

No copyrighted material, including figures, tables, or other content, from other sources has been reproduced in this manuscript.

## Supporting information


**Table S1** List of considered terms

## Data Availability

The data that support the findings of this study are available from the corresponding author upon reasonable request.
