# Supplementary material for: Inflammatory/Immune Adverse Events in Chronic Myeloid Leukemia Patients During Treatment With Bosutinib
Source: Cancer Med. 2025 Feb 5;14(3):e70580. doi: 10.1002/cam4.70580 (PMC11799592; doi:10.1002/cam4.70580)
Supplement: Supplementary file 1 — Table S1 List of considered terms [file CAM4-14-e70580-s001.docx]

| Arthralgia | Hypochromic area | Osteoarticular pain |
| --- | --- | --- |
| Articular pain | Horton’s arteritis | Osteomuscular pain |
| Bone pain | Hypothyroidism | Psoriasis |
| Diffuse pain | Joint pain | Sarcoidosis |
| Dry eyes | Joint stiffness | Subskin nodules |
| Erythema nodosum | Myalgia | Skin nodules |
| Erythematous papules | Muscular pain | Temporal arteritis |
| Erythema temporal area | Muscle pain | Temporal arteries pain |
| Giant cell arteritis | Musculoskeletal pain | Ulcer temporal area |
| Pleural effusion | Pericardial effusione | Fever |
| Agitation | Delirium | Depression |
| Hallucinations | Irritability | Mania |
| Personality change | Restlessness | Psychosis |
| Raynaud’s phenomenon |  |  |

Table S1. List of considered terms
